# Pain Retrained: Outcomes Analysis of an Online, Interdisciplinary Chronic Pain Education Programme

**DOI:** 10.1002/ejp.70288

**Published:** 2026-05-04

**Authors:** Maura McCarron, Francis Agnew, Claire Briggs, Jason Brooks, Martin Dempster, Danielle Rainey, Kevin E. Vowles

**Affiliations:** ^1^ School of Psychology, Queen's University Belfast Belfast UK; ^2^ Belfast Centre for Pain Rehabilitation, Belfast Health and Social Care Trust Belfast UK; ^3^ Psychological Services Research and Development Centre, Belfast Health and Social Care Trust Belfast UK

**Keywords:** chronic pain, interdisciplinary, online education, telehealth, videoconference

## Abstract

**Background:**

Videoconferencing to deliver chronic pain education offers improved accessibility and scalability. However, evidence regarding such interventions is largely absent, thus it is necessary to assess their clinical value. This study evaluated the impact of a group‐based interdisciplinary chronic pain education programme, Pain Retrained, delivered via videoconferencing.

**Methods:**

This study employed a single‐arm longitudinal design with measures collected at baseline, post‐intervention, and 3‐month follow‐up. Six weekly online sessions of two hours each were delivered to groups as the first point of contact with a secondary care pain service. Changes were evaluated via multilevel Structural Equation Modelling. Indices of acceptability were assessed post‐intervention.

**Results:**

In total, 261 participants (62.1% female) completed Pain Retrained. There was sustained change through follow‐up for pain self‐efficacy and social functioning and post‐intervention only change for depression and pain‐anxiety. Change was non‐significant for pain intensity and pain interference. Effect sizes ranged from negligible to medium (range Cohen's *d* = 0.03–0.74). The majority of participants, 85.4% reported improved understanding of pain.

**Conclusion:**

There has been growing interest in advancing pain education through online modalities. This study shows that live, clinician‐led online pain education is feasible and acceptable for people with chronic pain, with high satisfaction and perceived utility. These data are among the first to examine the effectiveness of this approach in relation to clinical outcomes. While outcomes were mixed, reflecting the complexity of chronic pain and limits of education alone, these findings extend existing knowledge by demonstrating this approach offers an accessible and scalable adjunct to care.

**Significance:**

The expansion of digital health interventions has outpaced evidence supporting their use in chronic pain care. By providing longitudinal, practice‐based evidence from a novel large‐scale programme, this study provides timely evidence to guide integration of online education into care pathways. It informs service planning while identifying key targets for optimisation, including integration with behaviour change strategies. The work also establishes priorities for further research to test efficacy and mechanisms of action in digitally delivered pain care.

## 1 Introduction

Chronic pain, defined as pain persisting or recurring for more than three months, affects approximately one in four adults globally, representing a significant public health concern (Cohen et al. 2021; Treede et al. 2019; Zimmer et al. 2022). Over the past several decades, advances in pain science have led to a paradigm shift from reductionist biomedical models to more comprehensive biopsychosocial frameworks that better reflect the complexity of pain (Fordyce 1976, 1994; Odling‐Smee 2023; Raja et al. 2020).

Contemporary clinical guidelines advocate for interdisciplinary treatment approaches that address behavioural, cognitive, sociocultural, and neurophysiological factors (National Institute for Health and Care Excellence 2021; U.S. Department of Health and Human Services 2019). However, the implementation of these approaches has been limited by various barriers at both individual and societal levels (Burgess et al. 2024). A key barrier is the widespread public understanding of chronic pain as primarily a physical or structural issue, which often conflicts with current scientific models and contributes to misconceptions and mistrust (Ryan et al. 2024). Such outdated beliefs may foster maladaptive pain behaviours, reduce engagement with effective treatments, and increase the risk of poor outcomes (Munigangaiah et al. 2016). For example, despite good evidence supporting psychological interventions for chronic pain, it is known that patient engagement can be limited due to doubts about their legitimacy or relevance (Vlaeyen and Morley 2005; Yang et al. 2015).

Pain education aims to address these misconceptions by aligning patients' understanding of pain with current scientific models. For individuals who conceptualise their pain within a purely biomedical framework, recommendations involving movement, sleep, or emotional regulation may appear irrelevant or invalidating. Moseley (2019) notes that such advice can feel counterintuitive, reinforcing disengagement. Therefore, pain education may play a critical role in reframing these strategies as logical and evidence‐based components of treatment (Leake et al. 2021).

One of the potential advantages of pain education is that it can be delivered remotely, increasing access to specialised services (Smith et al. 2019). While online formats offer scalability and convenience, evidence regarding their effectiveness and optimal delivery remains limited, particularly in relation to real‐time interaction with interdisciplinary teams (Mariano et al. 2021). Addressing this gap is essential for maximising the impact and reach of online pain education interventions.

In the present study, we examined the effectiveness of an online, interdisciplinary, chronic pain education programme, called Pain Retrained, through a three‐month follow‐up. The programme was developed to provide early education and initial contact with the interdisciplinary team as the first step in the treatment pathway for chronic pain. It addresses limited access to accurate information while offering a scalable model with the potential for broader reach and improved patient engagement. The primary objective of the present analysis was to examine the effectiveness of the educational programme across key pain‐related outcomes and to assess patient satisfaction in a large sample of newly referred individuals completing the programme.

## 2 Method

### 2.1 Participants

This study took place in a Northern Ireland‐based secondary care pain service within the United Kingdom's National Health Service (NHS). Ethical approval was in place to use the service's clinical data for research purposes (NHS Research Ethics Committee reference number: 313982). Upon entering the service, all patients were provided with information in relation to research activity and asked to provide informed consent that their de‐identified data could be used.

In the present analysis, data from all individuals who completed the Pain Retrained programme in calendar year 2022 were used. Power analyses were conducted using G*Power (ver 3.1, Faul et al. 2009). Assuming a longitudinal model with three assessment points and a small effect size (i.e., *f* = 0.15), results indicated that a sample size of 146 would be sufficient to detect an effect at power = 0.80. Participants were English speaking adults (over 18 years) who had been referred for treatment of chronic pain. In total, data from 261 patients were included. Of these, 62.1% were female, 35.0% were male and 2.9% declined to provide details. Participant age averaged 49.7 years (SD = 12.0), and years of education averaged 13.7 years (SD = 3.3). Regarding ethnicity, 92.4% were White, 1.5% were Asian or Asian British and the remaining 6.1% declined to provide details. In terms of relationship status, 45.2% were married or in a registered civil partnership, 26.7% were single, 3.4% were separated, 7.5% were divorced, 4.1% were widowed and 13.1% declined to provide details.

Average pain duration was 13.1 years (SD = 10.3). The average number of pain locations was 5.2 (SD = 1.8) with only 4.1% of participants noting a single location. Descriptively, the most frequent pain locations were lower back (89.7%) and lower extremity (85.6%), followed by neck/upper back (76.3%), upper extremity (68.6%), head (36.5%), and abdominal (36.1%). Concerning prior pain treatments, 74.5% underwent physiotherapy, 46.4% received injections, and 23.6% had surgery. Among those who received these treatments, only a small percentage reported finding them beneficial, with 20.9%, 23.6%, and 10.6%, respectively.

### 2.2 Measures

Following referral to the service, patients were asked to complete the clinic's self‐report assessment battery via a secure online link. Information regarding demographic and pain history was collected, as were responses to several standardised questionnaires regarding pain experience and impact, and behavioural and psychosocial functioning. This battery was repeated at the conclusion of Pain Retrained and at a three‐month follow‐up assessment. A patient satisfaction survey was also completed at programme conclusion. The survey questions were (1) ‘Has attending the Pain Retrained course improved your understanding about chronic pain?’ and (2) ‘Have you felt benefit from attending the Pain Retrained course?’ Participants responded with a ‘Yes’ or ‘No’ response. Open‐ended qualitative questions were also included to inform future course refinement.

#### 2.2.1 Pain Interference

The Patient Reported Outcomes Measurement Information System (PROMIS) Pain Interference—Short Form 8a was used to assess the impact of pain on daily functioning (Amtmann et al. 2010). The measure includes eight items, ranging from 1 (not at all) to 5 (very much), which are summed and then converted to a t‐score (mean 50; SD: 10) with higher scores indicating greater pain interference. The PROMIS Pain Interference measure is widely used in chronic pain and has demonstrated strong psychometric properties (Askew et al. 2016).

#### 2.2.2 Social Role Functioning

The PROMIS Ability to Participate in Social Roles and Activities—Short Form 8a was used to evaluate ability to engage in usual social roles and activities (Hahn et al. 2010). As with the PROMIS Pain Interference measure, there are eight items which range from 1 (not at all) to 5 (very much), which are summed and converted to a t‐score (mean 50; SD: 10) with higher scores indicative of better social functioning. The PROMIS Social Role measure has established reliability and validity (Terwee et al. 2019).

#### 2.2.3 Pain Self‐Efficacy

The two‐item Pain Self‐Efficacy Questionnaire (PSEQ‐2; Nicholas et al. 2015) was used to assess confidence in carrying out activities while experiencing pain. Items are rated on a six‐point scale from 0 (not at all confident) to 6 (completely confident) with summed scores ranging from 0 to 12 and higher scores indicating higher confidence. The PSEQ‐2 has demonstrated excellent psychometric properties (Dubé et al. 2021).

#### 2.2.4 Depression

The Patient Health Questionnaire‐9 (PHQ‐9; Kroenke et al. 2001) was used to assess depressive symptoms. Items are rated on a three‐point scale from 0 (not at all) to 3 (nearly every day). The items are summed to yield a total score that ranges from 0 to 27, with higher scores indicating greater symptom severity. The PHQ‐9 is an established depression screening measure that has been used across diverse populations (Levis et al. 2019; Negeri et al. 2021).

#### 2.2.5 Pain‐Related Anxiety

The four item Pain Anxiety Symptoms Scale (PASS‐4; Vowles et al. 2024) was used to assess pain‐related anxiety. Items are rated on a six‐point scale from 0 (never) to 5 (always), with summed scores ranging from 0 to 20 and higher scores indicating higher pain‐related anxiety. The PASS‐4 has shown strong psychometric properties in terms of internal consistency and reliable relations with other measures of pain‐related functioning (Vowles et al. 2024).

#### 2.2.6 Pain/Discomfort

Item four of the EQ‐5D‐5L was used to assess present pain level. The EQ‐5D‐5L is a widely used health status instrument which measures health using five levels of severity in five dimensions, one of which is level of pain/discomfort today (Herdman et al. 2011). This item is rated from 1 (no pain or discomfort) to 5 (extreme pain or discomfort).

### 2.3 Pain Education Programme

As noted, Pain Retrained is a chronic pain education programme developed and delivered by an interdisciplinary pain team. It consisted of six sessions, each lasting two hours, delivered weekly over six consecutive weeks. These were conducted in real‐time via videoconferencing (VC) on Microsoft Teams, with 20–25 patients per group. For most, this served as the first point of contact with the service and therefore provided more timely access to a specialist team.

A full description of the programme is available in the Supporting Information, reported according to the Template for Intervention Description and Replication (TIDieR) checklist (Hoffmann et al. 2014). Briefly, programme content included information provision regarding biopsychosocial conceptualisations of the pain experience (i.e., cognitive, emotional, and behavioural aspects that may affect both experiences of pain and pain behaviour), the role of movement and exercise in pain rehabilitation, stress and sleep guidance, analgesic use and risks, and the evidence base in relation to treatments. Sessions were designed to be partially didactic, where the treatment provider provided structured information regarding the session topic, and partially interactive, including both focused discussion questions and time for group discussion. All participants were provided with an accompanying workbook that included educational slides as well as links to additional resources. For the treatment team, education fidelity was maintained by (a) manualisation of programme components and (b) regular team meetings to discuss the content of sessions and refine approaches to challenges. Provision was made for those for whom the online programme is unsuitable, predominantly those with access barriers including language and internet availability. These individuals were offered an in‐person version of Pain Retrained and their data were not included in the present analysis given its focus on online pain education.

### 2.4 Analytic Approach

Prior to evaluating longitudinal outcomes, data integrity screening procedures were conducted. These included assessment of the internal consistency of outcome measures, evaluation of missing data patterns, examination of variable distributions, and detection of outliers. We also calculated treatment effect size between pre‐ and post‐programme and between pre‐programme and three‐month follow‐up. Cohen's *d* values were calculated using the formula of Morris and DeShon (2002), which corrects for correlation in repeated measurements across different assessment points. Interpretation of effect size values was in line with Cohen's (1988) recommendations of none/negligible for values below 0.20, small for values 0.20 to 0.49, medium for values 0.50 to 0.79, and large for values 0.80 and above. Standard errors were calculated using the formula of Hedges and Olkin (1985) to determine the 95% confidence intervals for each effect size.

Multilevel Structural Equation Modelling (SEM) was used to evaluate change in outcome measures across the three assessment points in order to allow hierarchical nesting of longitudinal data. For these analyses, data were nested at two hierarchical levels: level 1: time—repeated assessment at pre‐programme (week 1), post‐programme (week 6), and three‐month follow‐up (week 19) and level 2: within‐participant change. In addition, a separate SEM was conducted to examine change between pre‐ and post‐programme in order to investigate effects that may have emerged immediately after the intervention but were not sustained at follow‐up. Data management and descriptive analyses were performed using the Statistical Package for Social Sciences, version 29 (IBM Corp 2022). All other analyses were done using Mplus, version 8.11 (Muthén and Muthén 2024).

## 3 Results

### 3.1 Data Integrity Evaluation

Internal consistency for the outcome measures was acceptable across all time points. Specifically, the range of Cronbach's *α* was pre‐intervention: 0.74–0.94, post‐intervention: 0.80–0.95, three‐month follow‐up: 0.86–0.96. There were no missing data at the pre and post intervention timepoints as the online clinical data collection system is set up to capture key information and reminds patients when there are missing data. There was attrition at the 3‐month follow‐up time point with 60% of participants (*n* = 156) returning the clinic's self‐report assessment battery. A maximum likelihood approach was used in SEM analyses to include all available data.

Distributions of observed variables were assessed through skewness and kurtosis. Values were within acceptable limits, with absolute values for skewness all < 1.3 and for kurtosis all < 1.3. Given these findings and the sample size, data were deemed approximately normal and suitable for analysis using robust maximum likelihood estimation. With regard to outliers, inspections of distributions indicated that the data for 26 individuals were univariate outliers on one or more dependent measures, including Social Role Functioning (*n* = 3), Pain Self‐efficacy (*n* = 6), Pain Interference (*n* = 1), Pain‐anxiety (*n* = 7), and Pain/discomfort (*n* = 12). A sensitivity analysis was conducted to assess the impact of outliers on outcomes and as their exclusion did not alter the direction or significance of the main findings all data was retained. Evaluation of Mahalanobis distance revealed no multivariate outliers.

Descriptive information for all study measures at baseline, post‐programme, and 3‐month follow‐up is presented in Table 1. Effect sizes (Cohen's *d*), are also presented, along with their corresponding 95% confidence intervals. As shown in the table, both Social Role Functioning and Pain Self‐efficacy improved at post‐programme and three‐month follow‐up in comparison to pre‐programme. Descriptively, the effect size was small across both time periods for Social Role Functioning and medium for Pain Self‐efficacy. For two of the four remaining measures, including Pain Interference, and Pain/discomfort, scores remained stable across the three time points with no substantial evidence of change and a negligible effect size for both time periods (i.e., Cohen's *d* < 0.20). For the final two measures, Depression and Pain‐anxiety, there was evidence of improvement at post‐programme with a loss of treatment effect at follow‐up. For Depression, effect size was medium at post‐programme and small at follow‐up. For Pain‐anxiety, effect size was small at post‐programme and negligible at follow‐up.

**TABLE 1:** Descriptive information.

Variable	Mean (SD)			Effect size (95% CI)	
	Baseline	Post‐program	3‐month follow‐up	Baseline/post‐program	Baseline/follow‐up
Pain interference	69.11 (5.75)	68.90 (5.95)	69.00 (6.10)	0.05 (−0.07 to 0.17)	0.03 (−0.09 to 0.15)
Social role functioning	33.43 (6.14)	34.67 (6.52)	34.77 (6.60)	0.24 (0.11 to 0.36)	0.26 (0.13 to 0.38)
Depression	17.95 (6.58)	14.27 (7.68)	16.76 (7.10)	0.61 (0.48 to 0.74)	0.20 (0.07 to 0.32)
Pain self‐efficacy	1.87 (2.16)	3.58 (2.98)	4.32 (3.07)	0.52 (0.39 to 0.65)	0.74 (0.6 to 0.87)
Pain‐anxiety	15.03 (4.31)	14.19 (4.49)	14.58 (4.42)	0.21 (0.08 to 0.33)	0.11 (−0.01 to 0.23)
Pain/discomfort today	3.07 (0.70)	3.01 (0.72)	2.97 (0.78)	0.09 (−0.03 to 0.21)	0.15 (0.03 to 0.27)

The multilevel SEM indicated a significant effect of time for two of six outcomes including Social Role Functioning, *B* (standard error: SE) = 0.06 (0.02), *p ≤* 0.01, and Pain Self‐efficacy, *B* (SE) = 0.13 (0.01), *p* ≤ 0.001. There was no effect for the remaining outcomes, all *B* < 0.01, all *p* > 0.15. The SEM analysis for pre‐ and post‐programme showed a significant effect for four of the six outcomes, including Social Role Functioning, *B* (SE) = 0.27 (0.07), *p ≤* 0.001, Depression, *B* (SE) = 0.08 (0.03), *p ≤* 0.001, Pain Self‐efficacy, *B* (SE) = 0.34 (0.04), *p ≤* 0.001, and Pain‐anxiety, *B* (SE) = 0.16 (0.05), *p ≤* 0.001, with no effect for Pain Interference and Pain/discomfort, *B* < 0.01, all *p* > 0.15. With regard to participant satisfaction, 85.4% reported improved understanding of pain and 76.8% reported gaining benefit from the programme.

## 4 Discussion

The Pain Retrained programme was developed to address several challenges in chronic pain treatment including lack of access to accurate information, limited reach, and restricted scalability. A key pragmatic strength of Pain Retrained was its ability to be offered to large groups of treatment‐seeking patients early in their chronic pain care. In this study, a total of 261 individuals completed Pain Retrained in 2022 as their initial intervention in a secondary care chronic pain service. The feasibility, accessibility, and capacity of the Pain Retrained programme therefore appear reasonable, with high patient satisfaction reported.

The present analysis was intended to evaluate the impact of Pain Retrained on key pain‐related outcomes. The individuals in this study were an unselected cohort of people with chronic pain presenting to specialty care in the NHS. Pain severity, duration, and impact were generally significant, and there had been multiple unsuccessful interventions including medications, injections, physiotherapy, and surgical procedures. Thus, it can be argued that the sample here was likely a typical chronic pain sample with significant clinical complexity (Kamper et al. 2015).

Overall, the analyses indicated sustained benefit through three‐month follow‐up for pain self‐efficacy and social functioning and transient benefit for depression and pain‐anxiety, which were present post‐programme but absent at three‐month follow‐up. Specifically, pain self‐efficacy demonstrated a medium effect size across both time points, while social functioning showed a small effect size. For depression and pain‐anxiety, there was evidence of short‐term improvement at post‐programme, followed by a decline in effect at follow‐up. Depression showed a medium effect at post‐programme that diminished to a small, not significant effect at follow‐up, while pain‐anxiety demonstrated a small effect initially that was not maintained over time. There was no benefit for current pain intensity or pain interference.

The modest and relatively transient benefit of pain education indicated here is in keeping with the broader literature which suggests that pain education alone yields temporary gains rather than durable behavioural change (Gauntlett‐Gilbert and Brook 2018). For example, Geneen et al. (2015) found that while educational interventions for adults with chronic pain improved pain‐related knowledge, there were generally only small, short‐term effects on pain intensity and disability. However, the pattern of results observed in this study offers insights to the role and potential value of education, particularly if offered early in the treatment pathway. The small but sustained improvement in social functioning observed is noteworthy, given that social isolation is frequently identified as a core challenge of living with chronic pain (Karos et al. 2020). The VC, group‐based format of the current education program may have helped to mitigate this isolation by enabling access and fostering a sense of shared experience among participants, factors that are increasingly recognised as important contributors to well‐being in chronic pain populations (Matthias et al. 2016; Ryan and McGuire 2016). Similarly, improvements in pain self‐efficacy, reflecting a greater perceived ability to manage one's condition, may serve as a foundation for later behavioural change. Notably, 85.4% of participants also reported an improved understanding of their condition. These findings are clinically and theoretically relevant, and suggest that meaningful changes may be catalysed by education, perhaps even in the absence of sustained symptom reduction.

Of note, while improvements in pain self‐efficacy were statistically significant they were well below thresholds typically associated with meaningful improvements in disability (Nicholas et al. 2015). In order to capitalise on these small gains, an education programme such as Pain Retrained should be embedded within a stepped‐ or stratified‐care model as there is a need for subsequent individually tailored, behaviourally focused interventions. Positioned as the first point of contact within the current secondary care service, the programme is designed to provide a shared biopsychosocial framework and prepare patients for subsequent components of the treatment pathway. Following completion of the programme in our service, patients progress to a comprehensive medical assessment and interdisciplinary rehabilitation programmes, depending on individual needs and goals. Although the standardised format of the education programme limits individual tailoring, this design reflects its role as a scalable entry‐level intervention. While the modest effects observed suggest that the core educational content could potentially be delivered more efficiently, qualitative findings indicate that the extended format was valuable (McCarron et al. 2026).

Our results ought to be considered in the context of several strengths and limitations. Interrogation of interventions in routine‐care settings is essential to understanding their practical utility (Deenik et al. 2020). In this context, the present study provides valuable effectiveness data that contributes to the evidence base for online pain education. The study cohort comprised unselected patients referred under standard clinical pathways, and thus reflects the broader population typically seen in specialised pain services. Patients in routine‐care settings frequently present with more complex and heterogeneous needs compared to those recruited for highly controlled clinical trials (Lim et al. 2021), which enhances the external validity of our findings. However, the cohort's lack of cultural and ethnic diversity may limit generalizability to more diverse populations. In addition, the study did not include a control or comparator group, limiting our ability to account for the influence of concurrent treatments or other confounding variables. Future research would benefit from the inclusion of a control group to strengthen causal inferences.

These findings can be considered within both the broader context of evolving models for chronic pain treatment and growing interest in digital health interventions. Over the past few decades, the field has progressively shifted from biomedical and unimodal approaches toward biopsychosocial, interdisciplinary models which emphasise functional restoration over symptom elimination (Fordyce 1982). In this context, the improvements seen here are not only consistent with the aims of contemporary pain care but may also signal the *mechanistic* role of education as a preparatory, foundational component within such frameworks. This view is supported in the literature (Moseley 2019) and in particular by the qualitative work of Robinson et al. (2016), who found that pain education can support a process of reconceptualization. This term refers to a cognitive reappraisal whereby individuals come to view chronic pain as a complex and multifaceted experience rather than simply a direct marker of tissue damage (Moseley 2007). If pain education is effective in catalysing reconceptualization, it would be logical to position such interventions early in the clinical pathway and ensure they are accessible to the widest possible target population. The online, group‐based format used in the present study offers an efficient and scalable means of achieving this goal.

Within the context of digital health interventions, the findings underscore the potential of VC delivery of pain education by interdisciplinary teams. Interest in remotely delivered pain interventions has grown rapidly, accelerated in part by the COVID‐19 pandemic and increasing demand for accessible, scalable care (Eccleston et al. 2020). Despite this development, there remains a notable gap in published outcome data on VC delivery of pain education by interdisciplinary teams (Booth et al. 2022; Walumbe et al. 2021). The present study contributes to an underexplored area of the literature by reporting outcomes, including follow‐up data, from a large cohort of individuals with complex chronic pain who participated in a video‐conferenced, interdisciplinary education programme. The format employed represents a meaningful contribution to the evolving landscape of digitally delivered clinical education and may have important implications for service design, particularly in demonstrating the feasibility of delivering structured education and interdisciplinary expertise at scale, early in the care pathway.

Finally, these data invite a broader reflection on prevailing evaluation practices within the field of pain education. The pattern of results in the present study, alongside findings from previous literature, suggests that the primary role of education is *preparatory*, aimed at enhancing psychosocial functioning and understanding of pain rather than directly reducing symptoms or disability. As such, outcome measures based on expectations of symptom change may be of limited value and future evaluations would benefit from incorporating measures that more accurately reflect the role of education. Currently, there appears to be a lack of established measures designed specifically for this purpose although the Patient Activation Measure (Hibbard et al. 2004) may offer a useful proxy. In addition, qualitative methodologies could provide valuable insights into patients' evolving beliefs and intentions, offering a deeper understanding of how education influences their journey through care (Kazdin 2007).

In conclusion, there is significant demand for healthcare services to better address the needs of people living with chronic pain. Pain Retrained is a novel, VC‐delivered, interdisciplinary pain education programme which has improved accessibility and capacity in a secondary care pain service. This study provides preliminary evidence regarding the potential impact of this approach on clinical outcomes. While some effects were transient and others appeared sustained through three months, the absence of a control group limits the strength of causal inferences. Notably, 85.4% of participants reported improved understanding, reinforcing the value of education as a foundational component of treatment. Future iterations could benefit from including outcomes related to treatment readiness and qualitative process evaluations may be valuable in unpacking these complex factors. Overall, the findings suggest that offering online live, interactive, and interdisciplinary education to people with chronic pain is feasible and warrants further controlled evaluation.

## Author Contributions

This study was designed by M.M., K.E.V. and J.B. M.M. and K.E.V. performed the analyses, and all authors critically evaluated the results. M.M. wrote the first draft of the manuscript, which was edited by all authors. All authors have approved the final version of the manuscript.

## Funding

This research was conducted with the support of PhD funding from the Northern Ireland Department for the Economy.

## Conflicts of Interest

The authors declare no conflicts of interest.

## Supporting information


**Data S1:** ejp70288‐sup‐0001‐supinfo.docx.

**Appendix S1:** ejp70288‐sup‐0002‐AppendixS1.docx.
