# Supplementary material for: Pain Retrained: Outcomes Analysis of an Online, Interdisciplinary Chronic Pain Education Programme
Source: Eur J Pain. 2026 May 4;30:e70288. doi: 10.1002/ejp.70288 (PMC13138362; doi:10.1002/ejp.70288)
Supplement: Supplementary file 1 — Data S1: ejp70288‐sup‐0001‐supinfo.docx. [file EJP-30-0-s002.docx]

Description of Pain Retrained in accordance with the Template for Intervention Description and Replication (TIDieR) checklist (Hoffmann et al., 2014).

**Rationale and goals of intervention**

The Pain Retrained programme is a group-based pain education intervention. It is grounded in biopsychosocial and cognitive-behavioural models of chronic pain, and is designed as the initial point of contact for most patients referred to the specialist pain service, providing timely access to evidence-based education and contact with the clinical team. It provides patients with foundational knowledge and cognitive frameworks for understanding their pain experience.

**Intervention Delivery**

The programme comprises six weekly sessions, each lasting two hours and delivered online via Microsoft Teams. Sessions are conducted in groups of approximately 20 to 25 patients and are co-facilitated by two clinicians. There is representation from across the interdisciplinary team which consists of a clinical psychologist, medical doctor, occupational therapist, physiotherapist and specialist pain nurses. Each session includes both didactic and interactive components: clinicians present structured content relevant to that week’s topic, followed by facilitated group discussion to consolidate learning and encourage reflection. Interaction is supported through live discussion and chat functionality, with patients encouraged to share experiences and perspectives.

**Procedures and Materials**

Session content includes an introduction to the biopsychosocial model of pain, including cognitive, emotional, and behavioural contributors to pain experience and behaviour; the role of movement and exercise in pain management; guidance on sleep, stress and flare management; principles of medication use and associated risks; and a review of the evidence base underpinning common treatment approaches. All participants receive a structured workbook that includes slides from each session, summaries of key points, reflective exercises, and links to additional trusted resources. The programme content is standardised and not individually tailored, though facilitators may adjust pacing and emphasis based on group discussion. Facilitators are trained in the delivery of the programme and follow a manualised facilitator guide. Fidelity to the manual is supported through weekly interdisciplinary meetings where the delivery team reviews session experiences and addresses implementation challenges. While no formal fidelity scoring is used, co-facilitation and regular supervision help ensure consistency in delivery. No modifications to the programme were made over the course of the study period.
